# Anger and health risk behaviors

**Published:** 2010-11-25

**Authors:** ML Staicu, M Cuţov

**Affiliations:** ‘Carol Davila’ University of Medicine and Pharmacy BucharestRomania

**Keywords:** anger, hostility, coronary disease, bulimia nervosa, diabetes, car accidents

## Abstract

The present paper makes a research about negative effects of anger and hostile conduct on peoples' health status. We have studied scientific articles published between 2000 and 2010, that did not contradict our initial assumption. The literature demonstrates that anger, wheatear suppressed or expressed, can determine various diseases, it can influence the conduct of people suffering from bulimia nervosa or it can be the cause of the growing number of car accidents.

In order to avoid these risks, the intervention should not be limited to medication, but it should also involve a psychological help that should insist on ways of dealing with anger  without exposing the person to any kind of risk for his health or wellbeing.

## Introduction

Since antiquity, people have been aware of a harmful association of anger with health. Buddhism actually refers to this as one of the Three Poisons of the Mind, along greed and foolishness. In the psychosomatic field, anger and hostility benefited from an overwhelming attention, being considered health risk behaviors [1]. 

The scientific literature makes the distinction between hostility anger and aggression. Hostility is typically described as a negative attitude or cognitive trait directed toward others, anger as an emotional state that consists of feelings that vary in intensity from mild irritation or annoyance to intense fury, and aggressiveness as a verbal or physical behavioral pattern manifest in yelling, intimidation or physical assaults [1].

The present paper presents some of the health risks that hostility, anger and aggressiveness could cause–from coronary heart diseases, diabetes to bulimic behaviors and road accidents. Nowadays, anger manifestations are more and more obvious, strongly affecting interpersonal relationships. The scientific literature has shown a very big interest for the health risk involved by these behaviors and their manifestation should not be neglected by patients or therapists, because they can produce negative outcomes on health. 

## Health risks 

### Coronary heart diseases 

Goldel Hill et al. (2006) warn of the act that there are proofs that establish a positive association between negative emotions and conditions such as atherosclerosis or coronary heart disease (CHD). Anger can have a direct impact upon cardiovascular diseases through the HPA axis and the sympathetic nervous system, their activation leading to an excessive liberation of corticosteroids and catecholamine. The liberation of such stress hormones can produce an avalanche of events, including hemodynamic and metabolic modifications, vascular problems, and disorders of the cardiac rhythm. Anger can also contribute to the adoption of an unhealthy lifestyle (smoking, consumption of high caloric aliments, alcohol and caffeine consumption)[5]. 

The expression of anger can be made in at least three ways: if the expression can be motivated by constructive reasons (in order to solve a problem), other types of anger can be motivated by deconstructive reasons– in order to justify somebody's feelings or to intensify somebody's state of anger. This is why some studies considered that anger increases the risk for CHD, and other discovered that in some situations anger manifestation can have a protective role. 

A research conducted in Canada and published in 2010 had the objective of identifying health risks associated with the three types of anger manifestation. The participants have been followed for 10 years and the results revealed that decreased constructive anger in men and increased destructive anger justification in men and women are associated with increased risk of 10–year incident of CHD [6]. Outward expression and the suppression of anger seem to be associated with prognosis in women that had already suffered from a coronary event[7].

Vella and Friedman (2009) warn that hostile individuals are exposed to cynical attitudes and they are mistrusted by others and this can lead to frequent anger experimentation and other associated behaviors. Situations requiring anger inhibition may be more prevalent in the daily life experiences of hostile individuals than encounters permitting anger expression. The tendency to suppress anger has been linked to more pronounced carotid arterial stiffness and intima–medial thickness, sub–clinical indices of coronary heart disease, compared to individuals rating high on anger expression. Some evidence suggests that hostile persons who inhibit their anger expression are more likely to develop significant coronary atherosclerosis than hostile individuals who express their anger [8]. 

Other studies also demonstrated that anger and hostility are associated not only with the risk of coronary disease (CHD) among healthy population, but also with a negative prognosis in the case of individuals suffering of these diseases. In 2009, Chida et al published a meta–analysis of 25 studies that had investigated the risk of CHD among healthy population and 19 studies that had analyzed individuals with existing coronary heart disease. The review suggested that anger and hostility were associated with CHD outcomes both in healthy and CHD populations. Intriguingly, the harmful effect of anger and hostility on CHD events in the healthy populations was greater in men than women, suggesting that men are more responsive to anger and hostility factors in relation to CHD [1].

Low social support and cynical hostility increase the risk of developing coronary atherosclerosis (CAD) and the mortality. A research conducted with the participation of 223 patients and published in 2000 had the objective of determining if the impact of social support, anger expression and hostility are correlated to increased CAD morbidity and mortality. The patients were asked to answer three self–report questionnaires: questions concerning emotional social support, the State–Trait–Anger–Expression Inventory (STAXI) and the Cook–Medley cynical hostility scale. The patients have been followed for 2 years. Out of the 223 patients, 162 had an angiographic follow–up made after 2 years. Patients with CAD and low emotional social support who express anger outwardly are at a highly increased risk of disease progression, independent of medication or other risk factors [9]. 

Also, there are researches that investigated the association between anger expression and hypertension. A research published in 2004 measured the behavioral reactions to both neutral and anger–evoking role–play interactions were in 26 hypertensive and 15 normotensive patients. Hypertensive patients were identified as having low social skills when dealing with social interactions. Behaviorally, this conclusion was confirmed by the fact that hypertensive patients used negative verbalizations during confrontational interactions. They used less visual contact and were rated as being less assertive than normotensive controls. The authors' conclusion was that hypertension was associated with a social skills deficit, revealed during the assertive expression of anger [10]. 

### Bulimic behavior

Negative emotions have been considered a causal factor for bulimic behavior. 

According to Engel et al. (2007), anger is one of the relative negative aspects in the case of bulimic patients and revealed that the relationship between negative emotions and eating disordered behaviors may partially depend upon personality variables such as impulsivity.

The explicative models of bulimia nervosa (BN) very often underlined the importance of personality. Some personality traits (perfectionism, neuroticism, obsessive–compulsiveness and impulsivity) have been considered important causal variables in models of BN. The impulsivity benefited from a special attention [11]. 

The study conducted by Engel and al. was made with the participation of 133 women who met DSM–4 criteria for BN, with an average age of 25.3, Caucasian and unmarried (the majority). This research confirmed the results of previous studies that had discovered that anger was associated with a bulimic behavior. Increased levels of mean antecedent anger were associated with increased likelihood of binge eating and vomiting. Also, the authors found that impulsivity moderated the relationship between anger variability and the likelihood to binge eat. Subjects high in impulsivity were more likely to binge eat as their variability of anger increased compared to those at medium or low levels of impulsivity. Those low in impulsivity appeared to respond in the opposite manner: as anger variability increased, likelihood of binge eating decreased [11].

Not surprisingly, the authors found that the likelihood of vomiting following a binge–eating episode is significantly increased in bulimic participants. Somewhat surprisingly, however, they found that binge eating following vomiting behavior is less likely to occur. This suggests that once participants engage in purging behavior, they were quite unlikely to binge eat for a considerable amount of time. This pattern of results might be explained by bulimic patients engaging in binge eating and vomiting behavior which frequently is followed by extended periods of other behaviors, such as dietary restriction or exercise [11].

### Diabetes

Anger is associated with developing type 2 diabetes via two potential mechanisms: [1] through its association with poor health behaviors, resulting in obesity and/or [2] activation of the sympathetic nervous system, which leads to an inflammatory response initiated by interleukin–6 (IL–6) (Black, quoted by Golden Hill et.al) [5].

The research published by Golden et al. in 2006 investigated the association between anger proneness predicted type 2 diabetes. Over 11,615 initially non–diabetic adults aged 48–67,  were subsequently followed for 6 years for the development of type 2 diabetes. There was no relation between overall trait anger and the subsequent risk of diabetes; however, individuals in the highest levels of trait anger temperament scores had a 34% increased risk of developing diabetes compared to those in the lowest levels. Just like in the case of CHD, the more chronic, intense, and explosive components of trait anger may be more important in diabetes risk. This relationship has been also explained by lifestyle factors, suggesting that anger temperament may have a greater influence on behavioral and/or physiological changes that lead to obesity and thus, to diabetes. There are several mechanisms by which anger temperament might lead to the development of type 2 diabetes. Chronic anger can lead to poor health behaviors leading to central adiposity and type 2 diabetes. In this study, individuals with higher anger scores were more likely to smoke and had higher caloric intake [5].

A second mechanism is via activation of the sympathetic nervous system as catecholamine excess can lead to alterations in insulin sensitivity and can stimulate a chronic inflammatory response initiated by IL–6 (Black, 2003, quoted by Golden Hill et al, 2006) [5].

### Driving anger

Anger was also associated with an increased risk of road accidents. Becoming angry whilst driving appears to be a relatively common event and Sullman et al. remind us that the research on this subject demonstrated that angry drivers engage in aggressive and dangerous behaviors. Research has also found significant relationships to exist between driving anger and crash related conditions, such as losing control of their vehicle, losing concentration and crashing in simulator studies [12].

### Adolescent

In adolescents, the two types of anger (anger expression and anger suppression) can be associated with adopting unhealthy lifestyle behaviors (lack of physical activity and consumption of alcohol, cigarettes and caffeine). The analyze of the results A from a study(2000), that investigated the case of 411 adolescents, showed that teenagers high in anger suppression reported consuming alcohol more frequently, spending fewer hours per week in aerobic activity, and being less physically active than their peers. Teenagers high in anger expression reported consuming more caffeinated soda and coffee [13]. 

## Conclusions

Because the scientific literature demonstrated the connection between anger, hostility and aggressiveness and various health risks, the intervention for healing or preventing these diseases should not only be pharmacological, but also psychological; therapists should insist on ways of managing these behaviors in order to prevent diseases and any kind of risk that they could involve. 
